# Unusual clear cell tumors of the jaws – clinical and histopathological considerations: A case report

**DOI:** 10.1186/1752-1947-2-290

**Published:** 2008-09-01

**Authors:** Giulio Gasparini, Roberto Boniello, Alessandro Moro, Francesco Federico, Federica Castri, Sandro Pelo

**Affiliations:** 1 Maxillo-Facial Surgery, Catholic University Medical School, Rome, Italy; 2 Department of Pathology, Catholic University Medical School, Rome, Italy

## Abstract

**Introduction:**

Clear cell neoplasms of the jaw are very infrequent and a review of the literature reports only isolated cases of metastatic renal clear cell carcinoma of the jaw.

**Case presentation:**

A 68-year-old man presented with an osteolytic lesion of the left hemimandible. The first diagnostic hypothesis was a third molar follicular cyst. Surgical treatment consisted of enucleating the lesion preserving the alveolar nerve and extracting of the retained tooth. Unexpectedly, the lesion presented as a solid.

**Conclusion:**

The authors report a case of a clear cell neoplasm involving the jaw in which histopathological exam presented an indeterminate histology. The histological characteristics of this tumor make it unique in the international literature.

## Introduction

Clear cell neoplasms of the jaw are very rare and a review of the literature reports only isolated cases of metastatic renal clear cell carcinoma of the jaw [1-3]. In the present work, we report a case of a clear cell neoplasm involving the jaw in which histopathological exam presented an indeterminate histology. The histological characteristics of this tumor make it unique in the international literature.

## Case presentation

**Figure 1:**
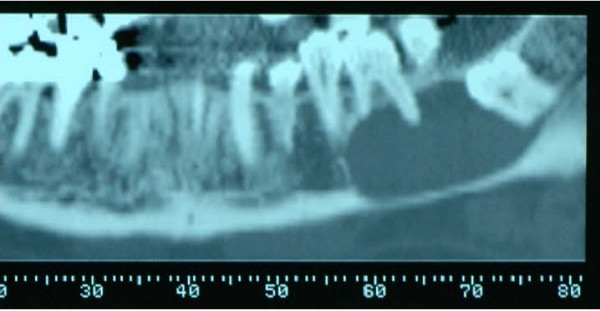
Pre-operative panoramic radiography.

A 68-year-old man presented to our center for treatment of an osteolytic lesion of the left hemimandible. The patient was completely asymptomatic and became aware of the lesion following a panoramic radiography. The lesion had spread into the linguo-vestibular thickness of the left hemimandible from the second premolar to the impacted third molar. Upon panoramic (Fig. 1) and computed tomography (CT) DentaScan investigation, the osteolytic lesion was seen to involve the first and second molar apex with amputation of the mesial root apex of the first molar. In spite of this, the teeth maintained pulp vitality. The lesion had clear edges and had eroded the lingual cortex.

The mandibular canal appeared to be eroded by the lesion, such that the alveolar nerve was circumscribed, even though the patient did not refer paresthesia. No intraoral or extraoral swelling was present.

The first diagnostic hypothesis was a third molar follicular cyst. A biopsy was not taken. Surgical treatment consisted of enucleating the lesion preserving the alveolar nerve and extracting the wisdom tooth. Unexpectedly, the lesion presented as a solid, sheet-like mass. At 6 months after resection, all teeth involved maintained vitality.

## Histopathological considerations

The histological specimen was sent to the Department of Pathology, Brigham and Women's Hospital and Harvard Medical School, Boston, USA for additional consultation. The lesion was classified as a clear cell tumor of undefined origin. It appeared as a grayish-white soft mass with a diameter of 3.8 cm. The tumor was composed of a monotonous, sheet-like proliferation of uniform cells having a clear cytoplasm and small nuclei; neither atypia nor pleomorphism could be detected and the mitotic index was within normal limits (2 mitoses/50 HPF).

**Figure 2:**
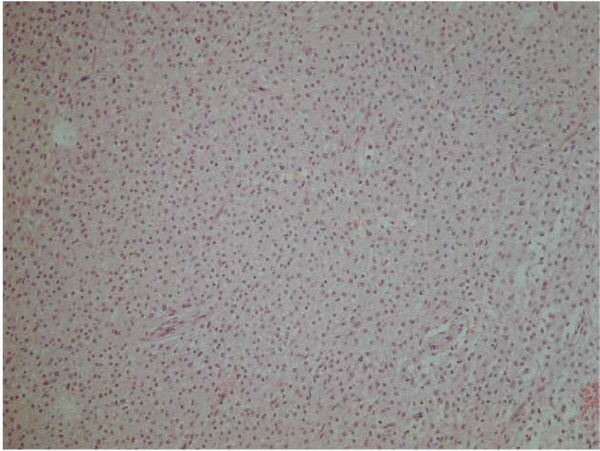
**Histological view.** 10× H&E – proliferation of uniform and monomorphic cells with small nuclei and clear cytoplasm; no atypia and no mitosis.

**Figure 3:**
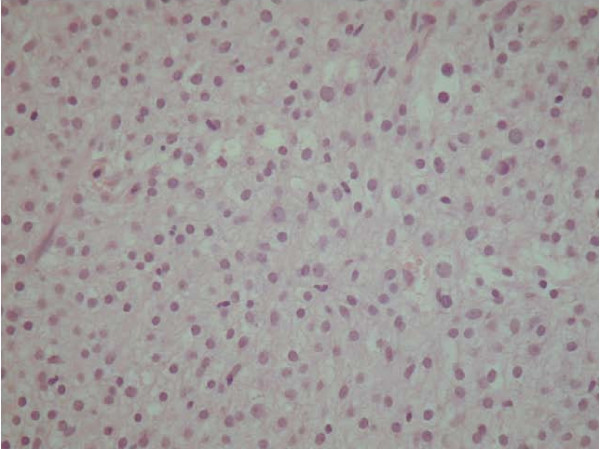
**Histological view.** 20× H&E – particular of Fig. 2.

The possibility of a rare intraosseous form of meningioma [4] was considered, as these neoplasms can assume different aspects. Immunostaining, however, did not substantiate this hypothesis. Ca 19.9, CD99, EMA and S100, which are positive in meningioma in 100%, 93%, 83%, 22% cases, respectively, were negative. However, the negativity for PanKeratin, HMB45 and LCA ruled out the possibility of a well differentiated epithelial, melanocytic or lymphocytic neoplasm, respectively. Desmin, CD68, SMA, GFAP, HLA-DR, CEA, C-erbB-2 and C-kit also stained negative. The only immunopositivity was for Vimentin (Figs. 2, 3).

The possibility of a metastatic renal lesion was considered, but both total-body CT scan and magnetic resonance were negative. A specimen was sent to an international specialist who also could not identify the origin of this neoplasm, and concluded that it should be considered an 'unclassified clear cell neoplasm'. The clinical course was to be carefully followed and a wait-and-see policy was adopted since no overt morphologic signs of malignancy were present.

## Discussion and conclusion

Clear cell neoplasms of the jaw are very rare. In a review of the literature, only isolated cases of metastatic renal clear cell carcinoma of the jaw have been described [1-3].

In the maxillo-facial area, clear cell odontogenic tumors have also been documented that are potentially aggressive and capable of multiple local recurrences and both locoregional and distant metastases. Management of this type of tumor should include wide en bloc resection and long-term follow-up [4]. Moreover, clear cells can be histologically present in ameloblastoma [5] and in rare intraosseous forms of meningioma [6] and might be considered in differential diagnosis of a jaw tumor.

In this patient, immunohistopathological exam presented an ambiguous pattern that did not permit a definitive diagnosis. Long-term follow-up will include clinical examination at 6-month intervals. In the follow-up, we decided to use a wait-and-see policy.

As this is an unidentifiable kind of neoplasm, it might represent a new type of lesion. We suggest considering this lesion as malignant, as long as it is impossible to find evidence of its benignity. That is why we highly recommend a follow-up with physical exam every 3 months in the first year, every 2–4 months during the second year, every 4–6 months from the third to the fifth year and every 6–12 months from the fifth year. The radiological exams should consist of CT of the maxillo-facial complex and neck with and without contrast agent every 6 months during the first year, alternating with echography of the neck every 3 months for the first year. From the third year, one echography every 6 months and CT every year [7]. We suggest treating the lesion as a malignant one in case of relapse with bone and soft tissue resection. For 'relapse', we mean every kind of lesion occurring next to the treated areas or every lymphnodal positivity with oncological characteristics. It is also mandatory to execute a lymphadenectomy in case lymph nodes are invaded.

## Competing interests

The authors declare that they have no competing interests.

## Authors' contributions

All of the authors were involved in examination of the patient as well as in writing and reviewing the manuscript.

## Consent

Written informed consent was obtained from the patient for publication of this case report and any accompanying images. A copy of the written consent is available for review by the Editor-in-Chief of this journal.
